# The decrease of glycolytic enzyme hexokinase 1 accelerates tumor malignancy via deregulating energy metabolism but sensitizes cancer cells to 2-deoxyglucose inhibition

**DOI:** 10.18632/oncotarget.24855

**Published:** 2018-04-10

**Authors:** Po-Lin Tseng, Chih-Wei Chen, Keng-Hsun Hu, Hung-Chi Cheng, Yuan-Ho Lin, Wen-Hui Tsai, Tain-Junn Cheng, Wei-Hsuan Wu, Chin-Wei Yeh, Chin-Chih Lin, Hui-Ju Tsai, Hao-Chun Chang, Jiin-Haur Chuang, Yan-Shen Shan, Wen-Tsan Chang

**Affiliations:** ^1^ Graduate Institute of Clinical Medical Sciences, College of Medicine, Chang Gung University, Taoyuan 302, Taiwan; ^2^ Division of Hepato-Gastroenterology, Department of Internal Medicine, Kaohsiung Chang Gung Memorial Hospital and Chang Gung University College of Medicine, Kaohsiung 833, Taiwan; ^3^ Institute of Clinical Medicine, College of Medicine, National Cheng Kung University, Tainan 701, Taiwan; ^4^ Department of Surgery, Chi Mei Foundation Medical Centre, Tainan 710, Taiwan; ^5^ Department of Occupational Safety and Health/Institute of Industrial Safety and Disaster Prevention, College of Sustainable Environment, Chia Nan University of Pharmacy and Science, Tainan 717, Taiwan; ^6^ Department of Biochemistry and Molecular Biology, College of Medicine, National Cheng Kung University, Tainan 701, Taiwan; ^7^ Institute of Basic Medical Sciences, College of Medicine, National Cheng Kung University, Tainan 701, Taiwan; ^8^ Department of Paediatrics, Chi Mei Foundation Medical Centre, Tainan 710, Taiwan; ^9^ Department of Neurology and Occupational Medicine, Chi Mei Foundation Medical Centre, Tainan 710, Taiwan; ^10^ Department of Pediatric Surgery, Kaohsiung Chang Gung Memorial Hospital–Kaohsiung Medical Center, Kaohsiung 833, Taiwan; ^11^ Department of Surgery, College of Medicine, National Cheng Kung University, Tainan 701, Taiwan

**Keywords:** glycolysis, hexokinase 1 (HK1), hexokinase 2 (HK2), Warburg effect, tumor metastasis

## Abstract

Malignant tumors often display an aberrant energy metabolism that relies primarily on glycolysis to produce adenosine triphosphate (ATP) the so-called Warburg effect or aerobic glycolysis. Thus, the elucidation of this energetic alteration in malignant tumors is important in the search for more effective therapeutics against malignant cancers, the most deadly human disease. To investigate whether attenuated glycolytic activity modulates tumor progression, the effects of silencing the first and rate-limiting glycolytic enzyme hexokinase (HK) isozymes HK1 and HK2 were examined. There was an inverse correlation between the expression of HK1 and HK2 in human cancer cells. In cervical carcinoma cells, the HK1 but not HK2 knockdown induced a phenotypic change characteristic of epithelial-mesenchymal transition, which accelerated tumor growth and metastasis both *in vitro* and *in vivo* analyses. Notably, the silencing of HK1 disrupted aerobic respiration and increased glycolysis, but it had no effect on ATP generation. These metabolic changes were associated with higher HK2 and lactate dehydrogenase 1 expression but a lower citrate synthase level. Particularly, the HK1 knockdown induced aberrant energy metabolism that was almost recapitulated by HK2 overexpression. Moreover, the HK1-silenced cells showed strong glucose-dependent growth and 2-deoxyglucose (2-DG) induced cell proliferation inhibition. These results clearly indicate that the silencing of HK1, but not HK2, alters energy metabolism and induces an EMT phenotype, which enhances tumor malignancy, but increases the susceptibility of cancer cells to 2-DG inhibition. In addition, this work also suggests that the glycolytic inhibitors should be used only to treat cancers with elevated glycolytic activity.

## INTRODUCTION

Glucose is an essential molecule needed for all living organisms. Its aberrant metabolism causes diverse disorders, such as obesity, diabetes and cancer, in particular malignant tumors [1–3]. For instance, malignant cancer cells often display a considerable increase in glucose uptake and metabolism, which provide a basic mechanism for tumor detection using positron emission tomography imaging probed with 2-[^18^F]fluoro-2-deoxy-D-glucose [4, 5]. In contrast, depleted glucose induces oxidative stress and results in extensive apoptosis in cancer cells, but not in normal cells [6–9]. Thus, the elucidation of glucose metabolism in malignant tumors may be of particular interest in the search for more effective therapeutics against malignant cancers, the most deadly human disease.

The first step of glucose metabolism is catalysed by hexokinase (HK), which phosphorylates glucose to form glucose-6-phosphate (G-6-P), which serves as a substrate for ATP production and metabolite biosynthesis [10, 11]. In mammals, there are four HK isozymes (HK1 to HK4) with different tissue and organ distributions, and distinct enzyme kinetics and metabolic functions [12, 13]. For example, HK1 is predominantly bound to mitochondria and mainly involved in catabolism for ATP generation, whereas HK2 is located mostly in the cytoplasm and participates principally in anabolism for metabolite formation [14, 15]. In particular, both the HK1 and HK2 isozymes can interact directly with mitochondria via a voltage-dependent anion channel (mVDAC) that controls cytochrome c release and therefore regulates cell apoptosis [16–20]. Moreover, the biological function of mitochondria-bound HK1 and HK2 is believed to optimise the ATP/ADP exchange between glucose phosphorylation and aerobic respiration [16, 17, 19]. Thus, either HK1 or HK2 overexpression in tumors is thought to provide both a metabolic benefit and an anti-apoptotic capacity that give the cancer cells a growth advantage and increase their resistance to anticancer therapy. In contrast, HK2 activity inhibited by 3-bromopyruvate (3-BrPA) causes tumor cell death by activating mitochondria-mediated apoptosis signalling [18, 20, 21]. However, the functional roles or effects of both HK1 and HK2 in glucose metabolism and the malignant progression of cancers are not fully understood, especially when their expression levels are decreased or low.

Elevated glycolytic activity increases lactate production and results in the acidification of the tumor microenvironment [22]. An acidified extracellular matrix has been shown to activate gelatinase activity and induce cathepsin B secretion, which increase the invasiveness of cancer cells [23, 24]. For instance, glycolysis-accelerated tumor metastasis has been demonstrated in cervical [25], colorectal [26] and head and neck [26, 27] cancers. In contrast, the inhibition of glycolysis by lactate dehydrogenase A (LDH-A) knockdown diminishes tumorigenicity [28]. In addition, elevated respiratory activity by mitochondrial frataxin overexpression inhibits tumor growth [29]. As compared with normal cells, tumors cells rely mostly on the glycolytic pathway for ATP production, even in the presence of abundant oxygen, the so-called Warburg effect or aerobic glycolysis [30, 31]. This heavier reliance of cancer cells on glycolysis for energy generation provides a possibility for anticancer therapy: glycolysis inhibitors may target and kill tumor cells by inhibiting ATP generation. For example, three HK inhibitors have been shown to be possible as chemotherapeutic agents: 2-deoxyglucose (2-DG), lonidamine and 3-BrPA [32]. Therefore, to target the glycolytic pathway for cancer treatment, it is important to clarify whether attenuated glycolysis directly modulates tumor progression.

Tumor malignancy resulting from aberrant energy metabolism might be caused by a mitochondrial defect [33, 34], a hypoxic environment [1], oncogenic signals [35] or enzymatic dysfunction [36]. For instance, glycolysis can be elevated by the overexpression of glycolytic enzymes or glucose transporters (Gluts), including Glut-1 or Glut-3 [37]. In contrast, respiratory activity may be impaired by a defect in a TCA cycle enzyme such as succinate dehydrogenase (SDH) or fumarate hydratase (FH), resulting in the activation of hypoxia inducible factor 1 (HIF-1) which in turn upregulates the glycolytic pathway [38–41]. In addition, it has been shown that the loss of the respiratory enzyme citrate synthase (CS) alters energy metabolism from aerobic respiration to glycolysis through deregulation of the tumor suppressors p53/TIGAR (TP53-induced glycolysis and apoptosis regulator) and SCO2 (synthesis of cytochrome c oxidase 2) [42–44]. Although many studies have indicated that highly malignant tumors most often rely less upon aerobic respiration and more on glycolysis for ATP production than their normal counterparts, to date, there has been little definitive data to demonstrate this hypothesis directly. To explore whether attenuated glycolytic activity affects energetic metabolism and further modulates tumor progression, the first and rate-limiting glycolytic HK isozymes HK1 and HK2 were silenced, and the effects were analysed.

## RESULTS

### Knockdown of HK1 but not HK2 induces an EMT phenotype

**Figure 1:**
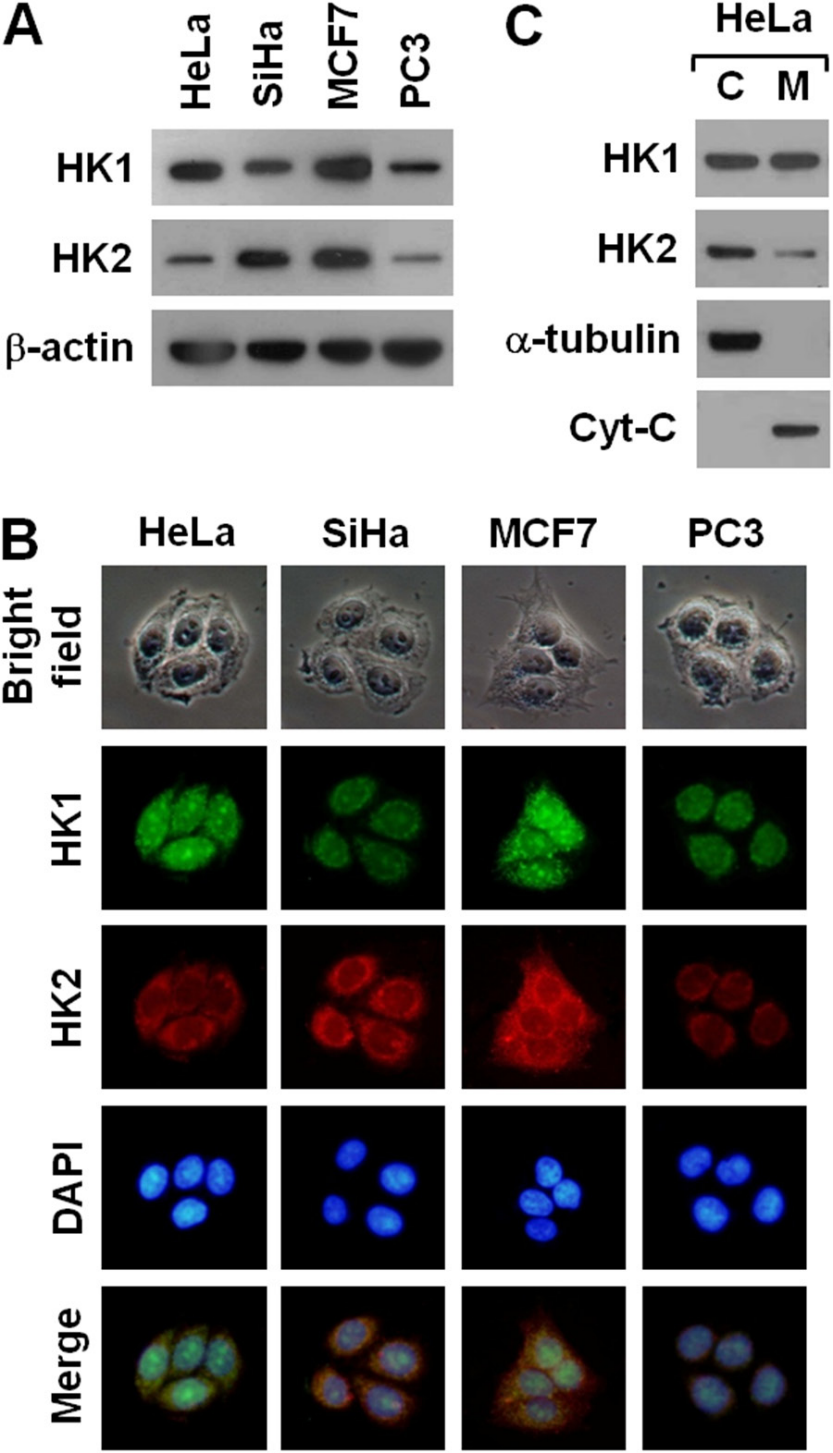
Human cancer cells display a strong inverse correlation between HK1 and HK2 expression **(A)** Western blotting of HK1 and HK2 in several cancer cell lines. Total protein extracts isolated from cells as indicated were blotted with antibodies specific for HK1, HK2 and β-actin. The β-actin level serves as the loading control for total proteins. **(B)** Immunofluorescent staining of HK1 and HK2 in various cancer cell lines. Cells as indicated were stained with antibodies specific for HK1 and HK2; nuclei were counterstained with DAPI. **(C)** Western blotting of HK1 and HK2 in cellular fractions isolated from HeLa cells. Cytosolic (C) and mitochondrial (M) isolates prepared from HeLa cells were blotted with antibodies specific for protein as labelled. The α-tubulin and cytochrome C (Cyt-C) serve as markers specific for the cytosolic and mitochondrial compartments, respectively.

To examine the connection between HK and tumorigenesis, we analysed HK1 and HK2 expression in a number of human cancer cells using Western blotting and immunofluorescent staining. Human cervical (HeLa and SiHa), breast (MCF7) and prostate (PC3) cancer cells expressed a substantial amount of HK1 and HK2 with variable detection levels (Figure 1A and 1B). Particularly, their expression patterns displayed a strong inverse correlation with high-low reciprocal levels. In addition, these two proteins were detected in both cytosolic and mitochondrial isolates with different distribution patterns. HK1 was distributed in both cytosolic and mitochondrial fractions, whereas the HK2 was detected mainly in the cytosolic fraction (Figure 1C). These results are consistent with previous studies, indicating that both HK1 and HK2 play important roles in energetic metabolism [16–21].

**Figure 2:**
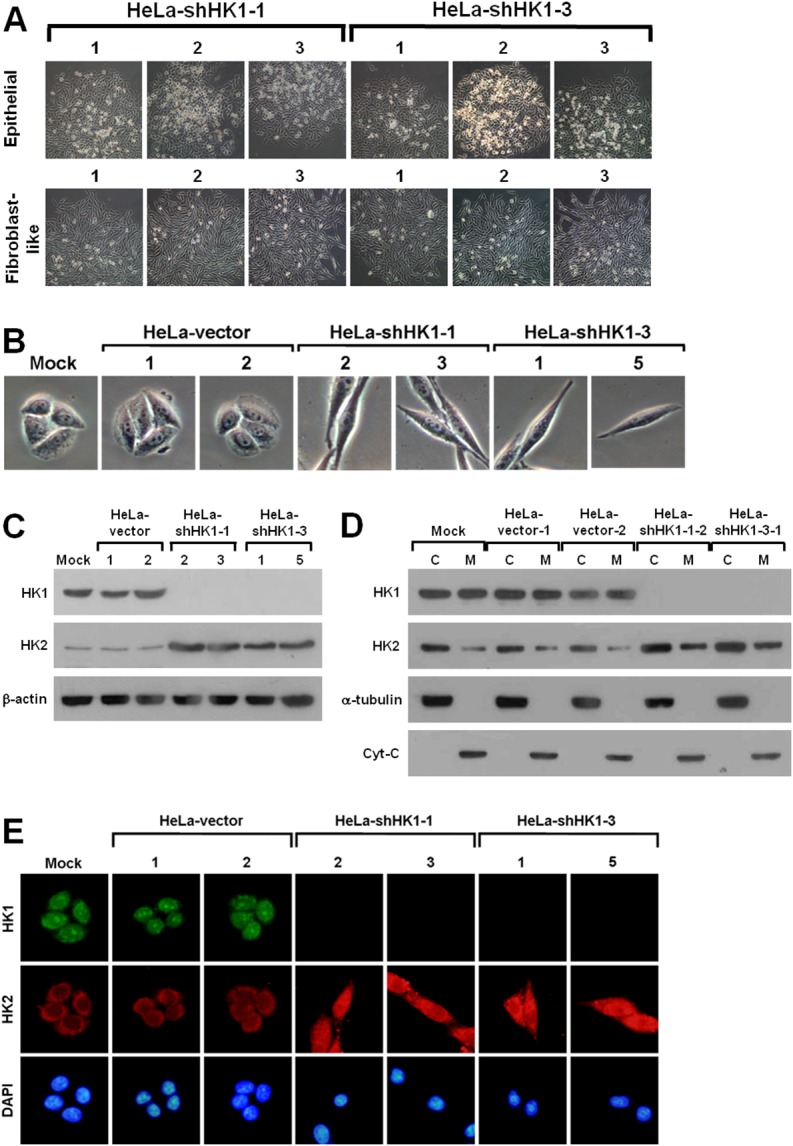
HK1 silencing induces a phenotypic switch **(A)** Colony morphology of shHK1-transfected cells. HeLa cells were first transfected with HK1 knockdown constructs (pshHK1-1 and pshHK1-3), and then selected for hygromycin B resistant colonies. Colonies were imaged and collected according to cell morphology. **(B)** Cell morphology of HK1-silenced cells. Four isolated and validated HK1-knocked down, mock and two vector-transfected cells were compared regarding cell morphology. **(C)** Western blotting of HK1 and HK2 in HK1-inhibited cells. Total protein extracts isolated from cells as indicated were blotted with antibodies specific for HK1, HK2 and β-actin. The β-actin level serves as a loading control for total proteins. **(D)** Western blotting of HK1 and HK2 in cellular fractions isolated from HK1-knocked down cells. Cytosolic and mitochondrial isolates prepared from cells as indicated were probed with antibodies specific for proteins as labelled. The α-tubulin and cytochrome C (Cyt-C) serve as markers specific for the cytosolic and mitochondrial compartments, respectively. **(E)** Immunofluorescent staining of HK1 and HK2 in HK1-silenced cells. Cells as indicated were stained with antibodies specific for HK1 and HK2; nuclei were counterstained with DAPI.

To analyse the functional role of HK in tumor progression, we established several stable HK1 or HK2 knockdown lines in human cervical carcinoma HeLa cells using the RNAi-mediated gene silencing approach. Two distinct hygromycin B-resistant colonies were generated during the selection of stable HK1-silenced cells (Figure 2A). One type of colony exhibited a particular fibroblast-like phenotype, whereas the other displayed the original epithelial morphology (Figure 2B). To examine the expression pattern of both HK1 and HK2 in these two colonies, Western blotting and immunofluorescent staining were carried out. As compared to the mock and vector-transfected cells, the fibroblast-like cells displayed almost complete knockdown of HK1 expression, while the epithelial cells exhibited a normal level of HK1 protein (Figure 2C and 2E). In addition, the HK1 protein was not detected in both cytosolic and mitochondrial isolates prepared from the fibroblast-like cells (Figure 2D). Notably, the HK1-knocked down cells displayed a considerable increase in HK2 expression as compared with the mock and vector-transfected cells (Figure 2C, 2D and 2E). These results reveal that silencing of HK1 induces a fibroblast-like morphology and simultaneously enhances HK2 expression. In contrast, only one type of hygromycin B-resistant colony with the original epithelial morphology was produced during the screening of stable HK2-silenced cells (Supplementary Figure 2A and Supplementary Figure 2B). To assess the expression of HK2 in selected clones, Western blotting and immunofluorescent staining were performed. The isolated cells exhibited specific and strong knockdown of HK2, and a considerable increase in HK1 expression as compared to the mock and vector-transfected cells (Supplementary Figure 2C, Supplementary Figure 2D and Supplementary Figure 2E). In addition, the HK2 protein was not observed in both cytosolic and mitochondrial fractions isolated from the selected cells (Supplementary Figure 2D). These results show that knockdown of HK2 does not induce any phenotypic alteration but at the same time promotes HK1 expression.

**Figure 3:**
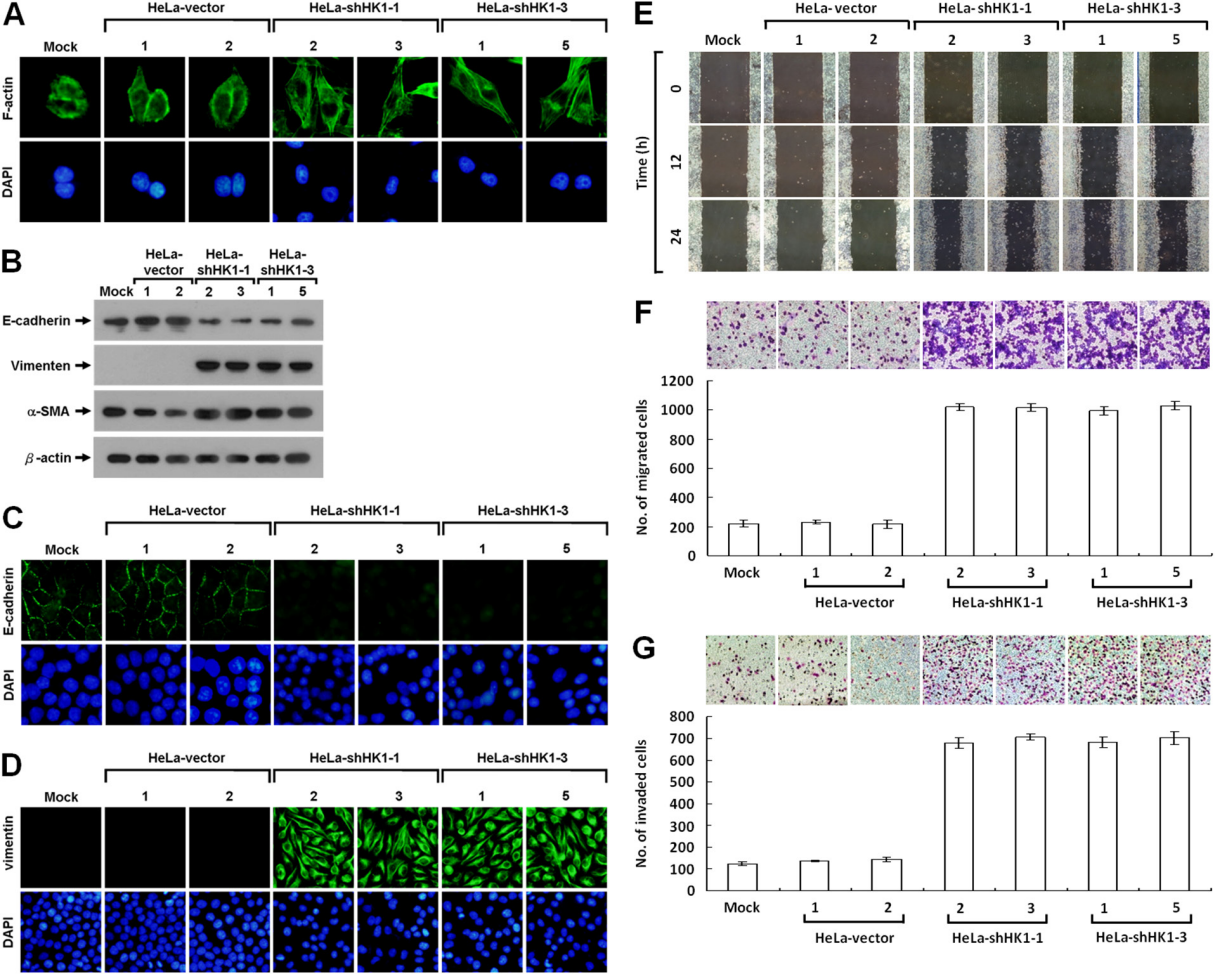
HK1 knockdown causes a typical EMT change and accelerates tumor cell motility **(A)** Fluorescence imaging of stress fibres in HK1-silenced cells. Cells as indicated were fixed and then stained with Alexa Fluor 488-conjugated phalloidin. **(B)** Western blotting of E-cadherin, vimentin and α-SMA in HK1-inhibited cells. Total protein extracts isolated from cells as indicated were blotted with antibodies specific for proteins as labelled. The β-actin level serves as the loading control for total proteins. **(C** and **D)** Immunofluorescent staining of E-cadherin and vimentin in HK1-knocked down cells. Cells as indicated were stained with antibody specific for E-cadherin or vimentin; nuclei were counterstained with DAPI. **(E)** Wound healing migration assay of HK1-inhibited cells. Cells as indicated were cultured until confluent, then the wound healing migration assay carried out; the wounds were imaged after incubation for various time periods as labelled. **(F)** Boyden chamber migration assay of HK1-knocked down cells. Cells as indicated were loaded into Boyden chambers, then migrated chemotactically for 6 h. The cells that migrated were stained, imaged and enumerated. **(G)** Matrigel invasion assay of HK1-silenced cells. Cells as indicated were seeded in invasion chambers, the invaded and penetrated chemotactically for 8 h. The cells that invaded were stained, imaged and enumerated. The plotted data are averaged from three independent experiments and the bars represent mean ± SD.

To analyse in detail the phenotypic change induced by HK1 silencing, we first examined the pattern of stress fibre F-actin formation using Alexa Fluor 488-conjugated phalloidin staining and further assessed the expression of EMT-specific markers, including E-cadherin, vimentin and α-smooth muscle actin (α-SMA) using Western blotting and immunofluorescent staining. Redistribution of the F-actin stress fibres was observed in the HK1-inhibited cells as compared with the mock and vector-transfected cells (Figure 3A). Furthermore, epithelium-specific E-cadherin was strongly downregulated, whereas the mesenchyme-specific markers (vimentin and α-SMA) were greatly upregulated after HK1 knockdown (Figure 3B). In addition, the expression patterns of both E-cadherin and vimentin were confirmed by immunofluorescent staining (Figure 3C and 3D). These results demonstrate directly that HK1 knockdown induces a typical EMT phenotype with a decrease in epithelial proteins and an increase in mesenchymal markers.

### EMT phenotype induced by HK1 knockdown greatly accelerates tumor malignancy

It is well-known that cancers, particularly epithelial carcinomas, undergo an EMT switch and then obtain the capacity to invade and metastasise, thereby increasing tumor malignancy [44–47]. Since the EMT phenotype induced by HK1 knockdown might enhance cancer cell migration and invasion, these behaviours were examined. Greater motility was seen in the HK1-silenced cells as compared to the mock and vector-transfected cells by the wound healing migration assay (Figure 3E). In addition, using the Boyden chamber migration assay, the number of HK1-knocked down cells that migrated was about five times higher than that observed in the mock and vector-transfected cells (Figure 3F). Moreover, approximately six times more HK1-silenced cells invaded in the Matrigel invasion assay compared to the mock and vector-transfected cells (Figure 3G). These results show that the EMT phenotype induced by HK1 knockdown increases tumor cell migration and invasion *in vitro*. In contrast, silencing of the HK2 did not alter the tumor cell motility as examined by the wound healing migration assay and quantified by both the Boyden chamber migration and Matrigel invasion assays (Supplementary Figure 3).

**Figure 4:**
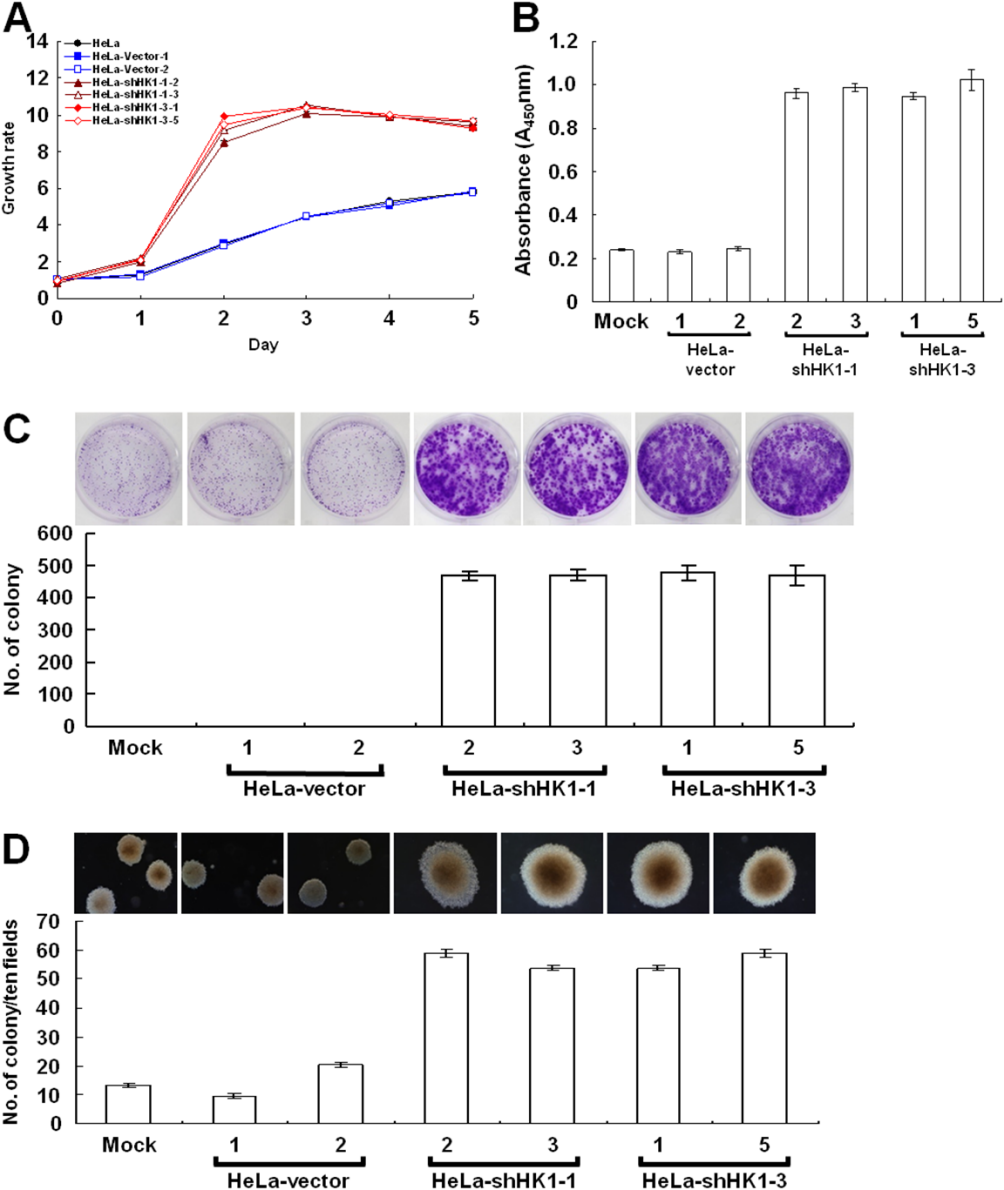
EMT phenotype caused by HK1 silencing increases cancer cell proliferation **(A)** MTT cell growth assay of HK1-knocked down cells. Cells as indicated were seeded in 96-well plates, incubated for various time periods as labelled, then the MTT cell growth assay was performed according to standard protocols. **(B)** BrdU incorporation assay of HK1-silenced cells. Cells as indicated were loaded into 96-well plates and incubated for 3 days, then the BrdU (colorimetric) cell proliferation ELISA was carried out according to the manufacturer’s procedures. **(C)** Colony formation assay of HK1-inhibited cells. Cells as indicated were cultured in 6-well plates for 6 days. Colonies were fixed, stained, imaged and enumerated. **(D)** Soft agar colony formation assay of HK1-knocked down cells. Cells as indicated were suspended in 0.35% top agar and overlaid on 0.7% bottom agar, then cultured in 6-well plates for 12 days. Colonies were directly imaged and enumerated. The plotted data are averaged from three independent experiments and the bars represent mean ± SD.

In the wound healing migration assay, the time needed for cells to grow to confluence was much shorter in the HK1-silenced cells than in the mock and vector-transfected cells. To examine the effect of HK1 knockdown on tumor cell proliferation, the MTT cell growth and 5′-bromo-2-deoxyuridine (BrdU) incorporation assays were performed. Greater cell growth was observed in the HK1-silenced cells as compared with the mock and vector-transfected cells (Figure 4A and 4B). In addition, to further assess the effect of HK1 knockdown on cancer cell growth, colony formation and soft agar clonogenic assays were carried out. Both anchorage-dependent and independent clonogenic growth was strongly enhanced, resulting in an increase in colony size and in the number of HK1-silenced cells (Figure 4C and 4D). These results reveal that HK1 knockdown promotes tumor cell proliferation *in vitro*. In contrast, silencing of HK2 did not influence cancer cell growth as analysed by both the MTT cell growth and BrdU incorporation assays, as well as in the anchorage-dependent and independent clonogenic growth assays (Supplementary Figure 4).

**Figure 5:**
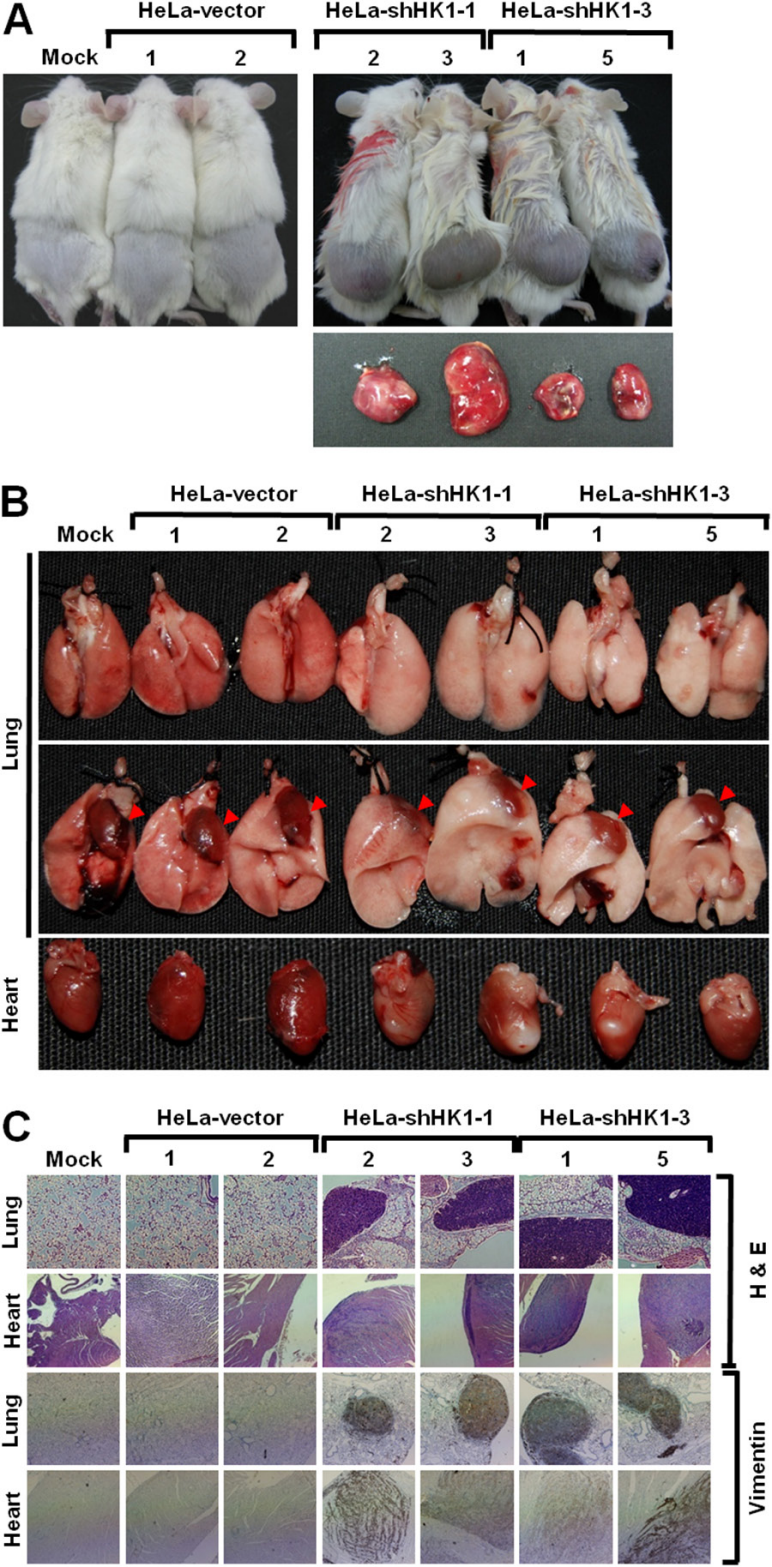
HK1 knockdown induced EMT switch accelerates tumor malignancy *in vivo* **(A)** *In vivo* cancer growth assay of HK1-silenced cells. Cells as indicated were subcutaneously inoculated into the back of NOD/SCID mice for 20 or 60 days. Mice were culled and tumors were excised and analysed. **(B)** *In vivo* cancer metastasis assay of HK1-inhibited cells. Cells as indicated were intravenously injected into the tail vein of NOD/SCID mice for 20 days. Mice were culled and examined for tumor metastasis. Red arrowheads indicate the heart. **(C)** Histological and immunohistochemical staining of the lung and heart in the *in vivo* tumor metastasis assay. Experiments were performed using H&E staining and an antibody specific for vimentin.

To analyse the effect of HK1 silencing on tumor malignancy *in vivo*, the cancer growth and metastasis of HK1-knocked down cells in the non-obese diabetic/severe combined immunodeficient (NOD/SCID) mouse were examined by a human tumor xenograft model. Fast growth and a large volume of tumor formation *in vivo* were observed in the HK1-silenced cells as compared to the mock and vector-transfected cells (Figure 5A and Table 1). This rapid growth was detected with only 1 × 10^5^ cells per mouse after subcutaneous inoculation of the HK1-knocked down cells for 20 days. Tail vein injection to assess *in vivo* tumor metastasis revealed greater and broader metastasis of HK1-silenced cells than the mock and vector-transfected cells (Figure 5B and Table 2). Metastasised lesions or foci of the HK1-knocked down cells were observed not only to the lung but also in the heart and mesentery tissues. In addition, the metastasised HK1-silenced cells displayed strong vimentin staining, while normal tissues, including the lung and heart, exhibited no vimentin staining (Figure 5C). Taken together, these results demonstrate that HK1 knockdown accelerates tumor malignancy, including increased cancer cell proliferation and metastasis.

**Table 1:** HK1 knockdown accelerates tumor cell growth *in vivo*

Cell line	Number of mice	Mean day for first measuring tumor size	Tumor size at 20 days (mm^3^)
**HeLa-mock**	**8**	**ND**	**0**
**HeLa-vector-1**	**8**	**ND**	**0**
**HeLa-vector-2**	**9**	**ND**	**0**
**HeLa-shHK1-1-2**	**9**	**10**	**1591.8 ± 579.9**
**HeLa-shHK1-1-3**	**8**	**10**	**1450.5 ± 450.2**
**HeLa-shHK1-3-1**	**9**	**10**	**1630.8 ± 739.8**
**HeLa-shHK1-3-5**	**8**	**10**	**1683.8 ± 709.1**

ND: no detectable tumor growth within 8 weeks after inoculation.

**Table 2:** HK1 silencing increases tumor cell metastasis *in vivo*

Organ	HeLa-mock	HeLa-vector		HeLa-shHK1-1		HeLa-shHK1-3	
		1	2	2	3	1	5
**Lung**	**0/6**	**1/6**	**0/6**	**3/6**	**4/6**	**4/6**	**4/6**
**Heart**	**0/6**	**0/6**	**0/6**	**3/6**	**2/6**	**2/6**	**2/6**
**Mesentery**	**0/6**	**0/6**	**0/6**	**3/6**	**3/6**	**5/6**	**3/6**

### HK1 knockdown impairs aerobic respiration but promotes glycolysis

**Figure 6:**
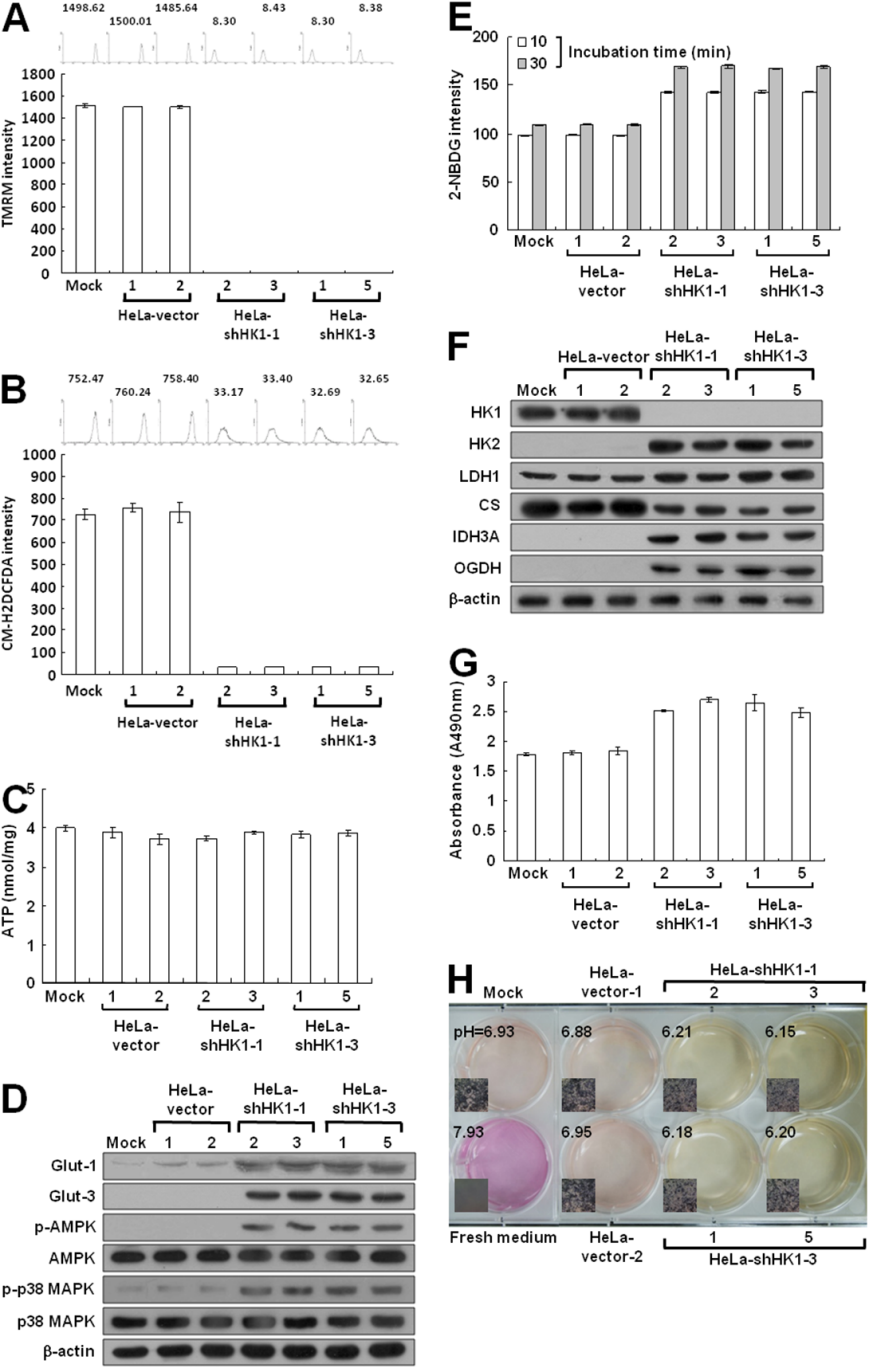
HK1 silencing induces severe defects in mitochondrial respiration but increases glucose uptake and promotes glycolytic activity **(A)** Δψ_m_ assay of HK1-knocked down cells. Cells as indicated were treated with TMRM and then fluorescence intensities were examined and analysed by a flow cytometer. **(B)** ROS assay of HK1-silenced cells. Cells as indicated were stained with CM-H2DCFDA and then fluorescence intensities were inspected and analysed by a flow cytometer. **(C)** ATP assay of HK1-knocked down cells. Total cell extracts prepared from cells as indicated were subjected to an ATP assay using the ATP Bioluminescence Assay Kit CLSII according to the manufacturer’s instructions. **(D)** Western blotting of Glut-1, Glut-3, p-AMPK and p-p38 MAPK expression in HK1-silenced cells. Total protein extracts isolated from cells as indicated were probed with antibodies specific for proteins as labelled. The β-actin level serves as the loading control for total proteins. **(E)** Glucose uptake assay in HK1-inhibited cells. Cells as indicated were treated with 2-NDBG and then fluorescence intensities were examined and analysed by a flow cytometer. **(F)** Western blotting of proteins involved in the TCA cycle and glycolysis in HK1-knocked down cells. Total protein extracts prepared from cells as indicated were blotted with antibodies specific for proteins as labelled. **(G)** Lactate dehydrogenase assay of HK1-silenced cells. Cells as indicated were subjected to a lactate dehydrogenase activity assay using the Lactate Dehydrogenase Assay Kit according to the manufacturer’s procedures. **(H)** pH measurement in conditioned medium of HK1-inhibited cells. Cells as indicated were cultured until confluent and then incubated in fresh medium. The color and pH value of the conditioned media were imaged and measured. The plotted data are averaged from three independent experiments and the bars represent mean ± SD.

To examine the effect of HK1 silencing on cellular energy metabolism, we first measured mitochondrial membrane potential (Δψ_m_) in HK1-knocked down cells using the tetramethylrhodamine methyl ester (TMRM) staining assay. The Δψ_m_ is established by complexes I, III and IV of the electron transport chain [11]. Therefore, the Δψ_m_ level corresponds with the activity of aerobic respiration [44, 48]. Strong staining was detected in the mock and vector-transfected cells; however, very weak or no staining was observed in the HK1-knocked down cells by the fluorescent probe, suggesting that HK1 silencing impairs aerobic respiration (Figure 6A). Because reactive oxygen species (ROS) are generated mainly from aerobic respiration, a decrease in aerobic respiration decreases the ROS level [11]. To confirm the impairment of aerobic respiration after HK1 knockdown, ROS was examined using the 5-(and-6)-chloromethyl-2',7'-dichlorodihydro fluorescein diacetate, acetyl ester (CM-H_2_DCFDA) staining assay. Very low or no CM-H_2_DCFDA staining was detected in HK1-silenced cells as compared with the mock and vector-transfected cells (Figure 6B). Together, these results suggest that HK1 knockdown impairs aerobic respiration and in turn decreases ROS formation.

To assess the effect of HK1 silencing on energy generation, ATP production was measured in HK1-knocked down cells by the firefly luciferase ATP assay. Surprisingly, the ATP level assessed in the HK1-silenced cells was similar to that measured in the mock and vector-transfected cells (Figure 6C). To further analyse the effect of HK1 knockdown on bioenergetic homeostasis, the phosphorylated AMP-dependent protein kinase (p-AMPK) level was examined in HK1-silenced cells using Western blotting. Notably, p-AMPK protein was elevated in the HK1-knocked down cells as compared to the mock and vector-transfected cells (Figure 6D). To confirm this effect in HK1-silenced cells, the phosphorylated p38 MAPK (p-p38 MAPK) level was further examined. The p-p38 MAPK protein was also increased in the HK1-silenced cells (Figure 6D). Taken together, these results indicate that HK1 knockdown impairs aerobic respiration and in turn disturbs bioenergetic homeostasis, resulting in an amplified AMPK/p38 MAPK pathway.

Because AMPK acts as a bioenergetic sensor, monitoring metabolic stresses such as hypoxia and respiratory deficiency [44, 49], any alteration that increases AMP or decreases the ATP level would upregulate AMPK activity and in turn promote glucose uptake^3^. Since cell glucose is imported by glucose transporters (Gluts), particularly Glut-1 and Glut-3 in tumors, their expression were examined in HK1-silenced cells by Western blotting. Glut-1 and Glut-3 levels were increased in HK1-knocked down cells as compared to the mock and vector-transfected cells (Figure 6D). In addition, increased glucose uptake was also detected in HK1-silenced cells using the 2-[N-(7-nitrobenz-2-oxa-1,3-diazol-4-yl)amino]-2 -deoxy-D-glucose (2-NDBG) staining assay (Figure 6E). These results also indicate that HK1 knockdown alters energetic metabolism and in turn enhances glucose uptake.

To validate the effect of energetic alteration on HK1-silenced cells, several glucose metabolism-related enzymes involved in both the TCA cycle and glycolysis were examined using Western blotting. Expression of key glycolytic enzymes HK2 and LDH1 were increased in HK1-knocked down cells as compared with the mock and vector-transfected cells (Figure 2C, 2D, 2E and 6F). However, expression of the first TCA cycle enzyme citrate synthase (CS) was decreased after HK1 silencing; in contrast, the expression of mitochondrial NAD^+^-specific isocitrate dehydrogenase α subunit (IDH3A) and oxoglutarate dehydrogenase (OGDH) was upregulated in HK1-knocked down cells. These results demonstrate that HK1 silencing deregulates glucose metabolism-related enzyme expression in both the TCA cycle and glycolysis. To confirm the effect of increased LDH1 level in HK1-silenced cells, lactate dehydrogenase activity was assessed. Consistent with increased LDH1 expression, the activity of the lactate dehydrogenase was enhanced in HK1-silenced cells as compared with mock and vector-transfected cells (Figure 6G). Since elevated lactate dehydrogenase activity could increase lactate production which in turn acidifies the conditioned medium, to examine the effect of increased lactate dehydrogenase activity, changes in the color and pH value of conditioned media were examined in HK1-knocked down cells. As compared to the mock and vector-transfected cells, a quick color change and lower pH value were observed in conditioned media prepared from HK1-silenced cells (Figure 6H). Taken together, these results reveal that HK1-silenced cells undergo a metabolic shift from aerobic respiration to glycolysis.

### Overexpression of HK2 alters energy metabolism but does not promote tumor malignancy

**Figure 7:**
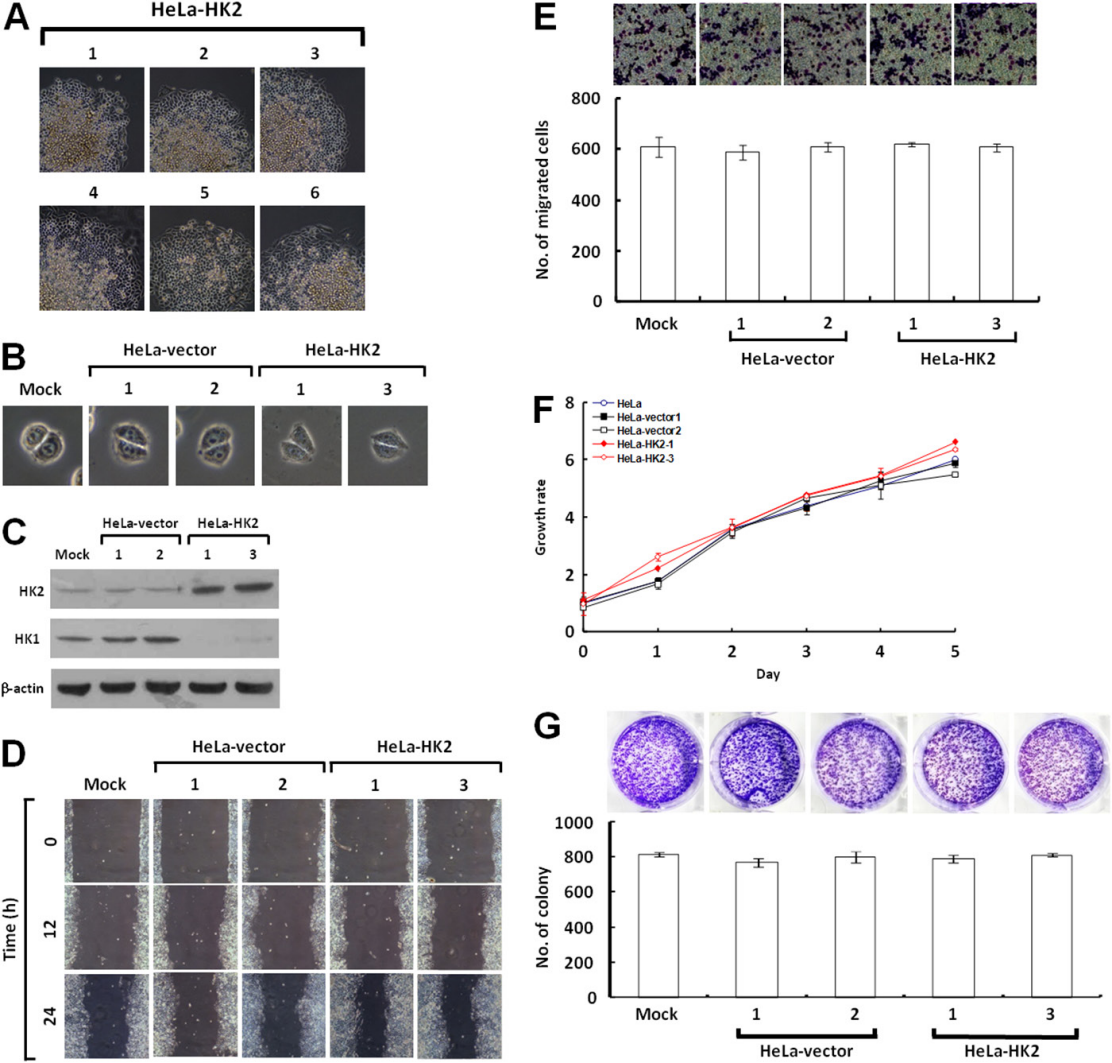
HK2 overexpression does not affect tumor cell motility and growth **(A)** Colony morphology of HK2-transfected cells. HeLa cells were first transfected with the HK2 expression construct, and then screened for G418 resistant colonies. Colonies were selected according to cell morphology and imaged. **(B)** Cell morphology of HK2-overexpressing cells. Two isolated and validated HK2-overexpressing, mock and two vector-transfected cells were compared in cell morphology. **(C)** Western blotting of HK2 and HK1 in HK2-overproducing cells. Total protein extracts prepared from cells as indicated were probed with antibodies specific for HK2, HK1 and β-actin. The β-actin level serves as the loading control for total proteins. **(D)** Wound healing migration assay of HK2-overexpressing cells. Cells as indicated were cultured until confluent, then the wound healing migration assay was carried out and the wounds were imaged after incubation for various time periods as labelled. **(E)** Boyden chamber migration assay of HK2-overproducing cells. Cells as indicated were loaded into Boyden chambers and migrated chemotactically for 6 h. The cells that migrated were stained, imaged and enumerated. **(F)** MTT cell growth assay of HK2-overexpressing cells. Cells as indicated were seeded in 96-well plates, incubated for various time periods as labelled, then the MTT cell growth assay was performed according to standard procedures. **(G)** Colony formation assay of HK2-overproducing cells. Cells as indicated were cultured in 6-well plates for 10 days. Colonies were fixed, stained, imaged and enumerated. The plotted data are averaged from three independent experiments and the bars represent mean ± SD.

**Figure 8:**
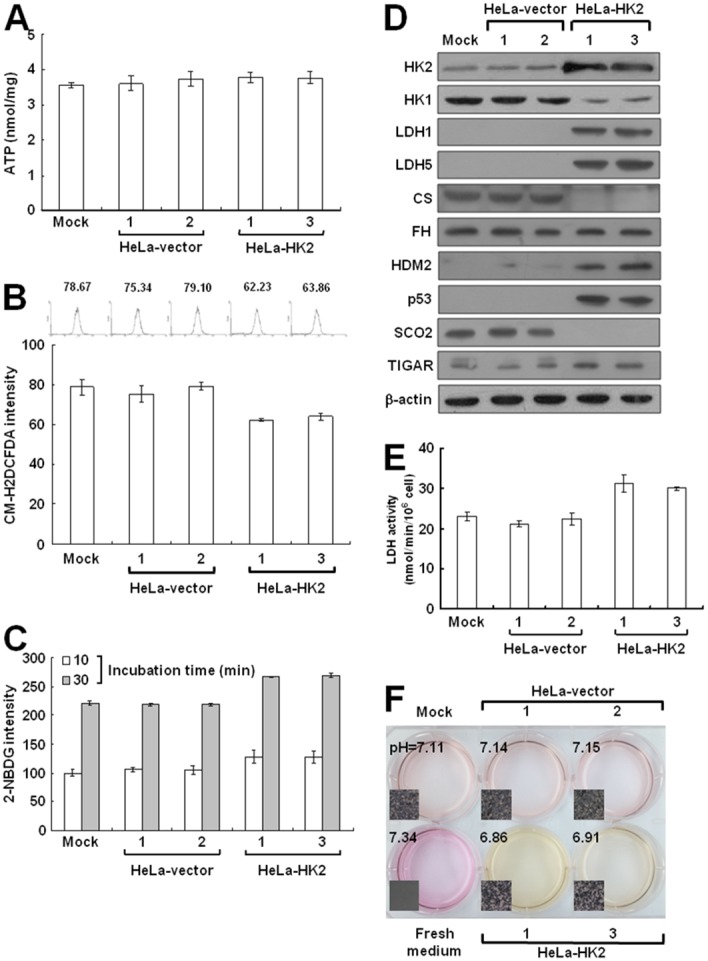
HK2 overproduction alters energetic metabolism **(A)** ATP assay of HK2-overexpressing cells. Total cell extracts prepared from cells as indicated were subjected to an ATP assay using the ATP Bioluminescence Assay Kit CLSII according to the manufacturer’s instructions. **(B)** ROS assay of HK2-overexpressing cells. Cells as indicated were stained with CM-H2DCFDA and then fluorescence intensities were inspected and analysed by a flow cytometer. **(C)** Glucose uptake assay of HK2-overproducing cells. Cells as indicated were treated with 2-NDBG and then fluorescence intensities were examined and analysed by a flow cytometer. **(D)** Western blotting of proteins participated in the TCA cycle and glycolysis in HK2-overexpressing cells. Total protein extracts prepared from cells as indicated were blotted with antibodies specific for proteins as labelled. The β-actin level serves as the loading control for total proteins. **(E)** Lactate dehydrogenase assay of HK2-overproducing cells. Cells as indicated were subjected to a lactate dehydrogenase activity assay using the Lactate Dehydrogenase Assay Kit according to the manufacturer’s procedures. **(F)** pH measurement in conditioned medium of HK2-overexpressing cells. Cells as indicated were cultured until confluent and then incubated in fresh medium. The color and pH value of the conditioned media were imaged and measured. The plotted data are averaged from three independent experiments and the bars represent mean ± SD.

To test whether the HK2 overexpression induced by HK1 knockdown plays a decisive role in energetic alteration, HeLa cells were transfected with an HK2 overproduction construct (pWZL-Neo-Myr-Flag-HK2) and cells with strong HK2 expression were selected. Only one type of colony with the original epithelial morphology was observed during screening of the stable HK2-overexpressing cells (Figure 7A and 7B). As compared with the mock and vector-transfected cells, HK2-overproducing cells indeed expressed substantial amounts of HK2 protein, but in contrast displayed a strong decrease in the HK1 level (Figure 7C). To examine whether HK2 overproduction affected tumor malignancy, cell migration and proliferation assays were carried out. The wound healing and Boyden chamber migration assays, as well as the MTT cell growth and colony formation assays revealed that both cell motility and growth in HK2-overexpressing cells were similar to the mock and vector-transfected cells (Figure 7D, 7E, 7F and 7G). However, HK2 overproduction promoted glycolytic metabolism, including increased glucose uptake and LDH activity and decreased the pH value of conditioned media (Figure 8C, 8E and 8F). In addition, the HK2-overexpressing cells also displayed strong LDH1 and LDH5 expression (Figure 8D). Moreover, HK2 overproduction caused deregulation of the p53/TIGAR and SCO2 pathways (Figure 8D). Taken together, these results indicate that HK2 overexpression does not fully phenocopy HK1-knocked down cells but indeed enhances glycolytic metabolism.

### HK1 knockdown increases the susceptibility to 2-deoxyglucose (2-DG) inhibition

**Figure 9:**
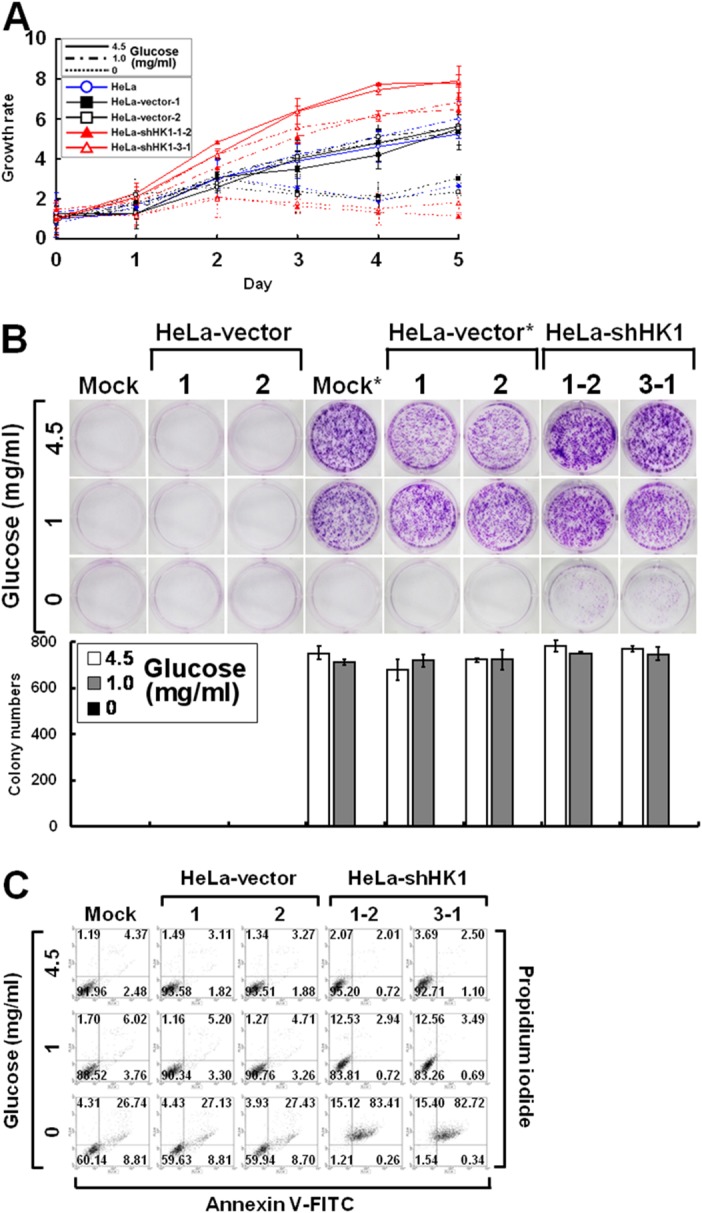
Silencing of HK1 enhances glucose dependence during cell growth **(A)** MTT cell growth assay of HK1-knocked down cells cultured in various glucose concentrations. Cells as indicated were seeded in 96-well plates with culture media containing 4.5, 1 and 0 mg/ml glucose. After incubation for various time periods, an MTT cell growth assay was performed according to standard protocols. **(B)** Colony formation assay of HK1-silenced cells cultured in different glucose concentrations. Cells as indicated were cultured in 6-well plates with media containing 4.5, 1 and 0 mg/ml glucose for 6 or 10 days. After incubation, colonies were stained, imaged and enumerated. ^*^ indicates incubation for 10 days. **(C)** Cell apoptosis assay of HK1-knocked down cells cultured in distinct glucose concentrations. Cells as indicated were grown in 6-well plates with media containing 4.5, 1 and 0 mg/ml glucose for 3 days. After incubation, cells were collected, fixed and subjected to a cell apoptosis assay using the FITC Annexin V Apoptosis Detection Kit I according to the manufacturer’s procedures. Fluorescence intensities of propidium iodide and annexin V-FITC stained cells were analysed using a flow cytometer. The plotted data are averaged from three independent experiments and the bars represent mean ± SD.

To examine the effect of increased glucose uptake on cell growth, the proliferation of HK1-knocked down cells cultured in medium with high (4.5 mg/ml), low (1 mg/ml) or no (0 mg/ml) glucose was assessed by the MTT cell growth and colony formation assays. As compared with the mock and vector-transfected cells, HK1-silenced cells grew rapidly in high and low glucose media, particularly in the high glucose medium. In the no glucose medium, however, they proliferated slowly for the first 48 h and then died in the MTT cell growth assay (Figure 9A). In addition, this enhanced cell growth was accompanied with increased colony number and size in high and low glucose media, but cells formed loose incomplete colonies in no glucose medium in the colony formation assay (Figure 9B). To examine this glucose depletion-induced cell growth inhibition, HK1-knocked down cells cultured in different glucose-containing media were analysed by propidium iodide and annexin V-FITC staining and flow cytometry. In high glucose medium, the HK1-silenced, mock and vector-transfected cells showed little evidence of cell death, including apoptosis and necrosis. In low or no glucose medium, in contrast, HK1-knocked down cells exhibited increased cell death compared to the mock and vector-transfected cells (Figure 9C). These results indicate that HK1-silenced cells display strong glucose-dependent cell proliferation.

**Figure 10:**
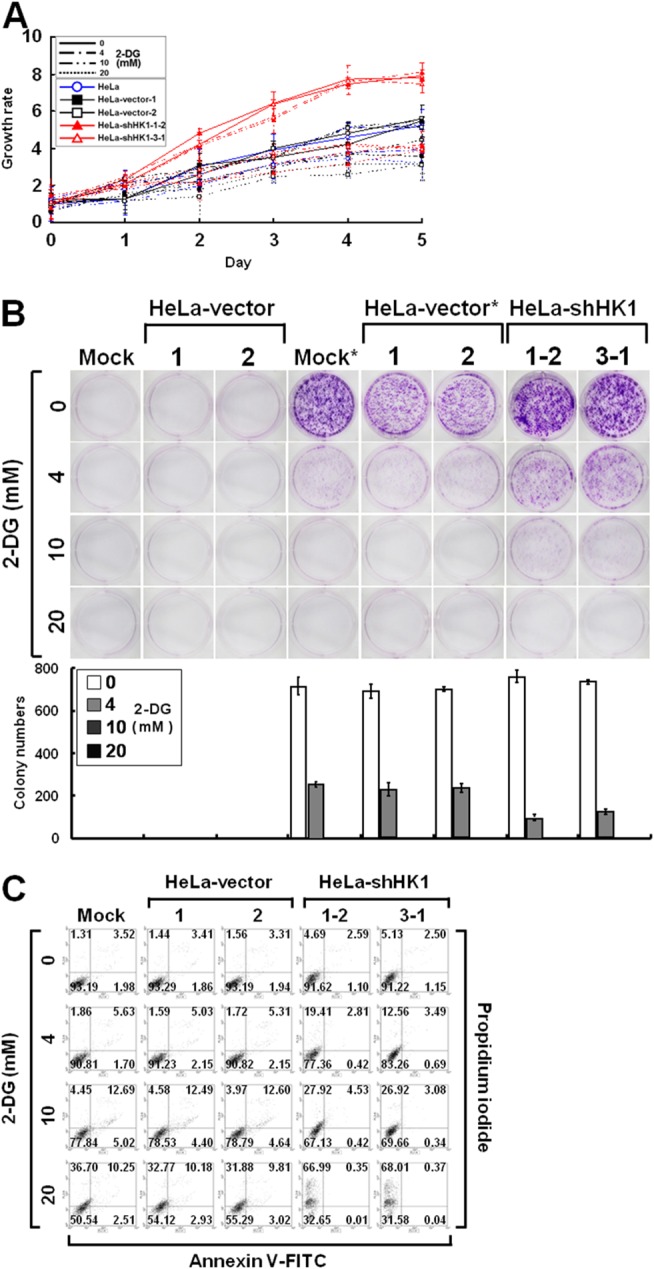
HK1 silencing increases the susceptibility to 2-DG inhibition **(A)** MTT cell growth assay of HK1-knocked down cells cultured in the presence of 2-DG. Cells as indicated were loaded in 96-well plates in the presence of various concentrations of 2-DG as labelled and then carried out MTT cell growth assay according to standard protocols. **(B)** Colony formation assay of HK1-silenced cells cultured in the presence of 2-DG. Cells as indicated were seeded in 6-well plates in the presence of different concentrations of 2-DG as indicated for 6 or 10 days. After incubation, colonies were stained, imaged and enumerated. ^*^ indicates incubation for 10 days. **(C)** Cell apoptosis assay of HK1-knocked down cells grown in the presence of 2-DG. Cells as indicated were cultured in 6-well plates in the presence of different doses of 2-DG as labelled for 3 days. After incubation, cells were harvested, fixed and subjected to a cell apoptosis assay using the FITC Annexin V Apoptosis Detection Kit I according to the manufacturer’s protocols. Fluorescence intensities of propidium iodide and annexin V-FITC stained cells were analysed using a flow cytometer. The plotted data are averaged from three independent experiments and the bars represent mean ± SD.

To examine the effect of glucose deprivation on cell proliferation, the growth of HK1-knocked down cells cultured in the presence of 2-deoxyglucose (2-DG), a non-metabolisable analogue of glucose, was assessed using the MTT cell growth and colony formation assays. As compared to the mock and vector-transfected cells, the HK1-silenced cells exhibited a strong response to 2-DG-induced growth inhibition in both a dose- and time-dependent manner (Figure 10A and 10B). To evaluate the effect of 2-DG induced growth inhibition, HK1-silenced cells cultured in the presence of 2-DG were analysed by propidium iodide and annexin V-FITC staining and flow cytometry. 2-DG induced cell death was higher in HK1-knocked down cells than in the mock and vector-transfected cells (Figure 10C). These results reveal that HK1 silencing results in increased susceptibility to 2-DG induced growth inhibition in malignant cancer cells.

## DISCUSSION

In this study, we first showed that HK1 but not HK2 knockdown induced a typical EMT switch in human cervical cancer cells. In addition, we demonstrated that this EMT change accelerated tumor malignancy, with increased cancer cell metastasis and proliferation observed using both *in vitro* assays and *in vivo* tumor xenograft models. Furthermore, we elucidated the possible underlying mechanism of this malignant progression induced by HK1 knockdown. In HK1-silenced cells, HK1 knockdown correlated with impairment of respiratory activity, which caused an alteration in bioenergetic homeostasis, and in turn increased glucose uptake via enhanced Glut-1 and Glut-3 expression. In addition, enhanced levels of the glycolytic enzymes HK2 and LDH1 were detected in HK1-knocked down cells; in contrast, reduced TCA cycle enzyme CS expression accompanied by increased expression of other respiratory enzymes was observed in HK1-silenced cells. Particularly, HK1 silencing induced alterations in energetic metabolism that were nearly recapitulated by HK2 overexpression and also observed in CS-knocked down cells [44]. Together, HK1 silencing not only induced a switch in energy metabolism from aerobic respiration to glycolysis, but also caused tumor malignancy, including increased cancer cell proliferation and metastasis.

Four HK isozymes have been identified with distinct tissue and organ distributions, as well as enzyme kinetics [12, 13]. Among these isozymes, both HK1 and HK2 play critical roles in promoting cell proliferation and survival in malignant cancers [16, 21, 50–53]. Overexpression of either the HK1 or HK2 has been detected in many tumors, including breast, colon and prostate cancers, cervical carcinoma, gastric adenoma, glioma and lymphoma [52, 53]. In this study, HK1 knockdown increased the HK2 level; in contrast, silencing of HK2 elevated HK1 expression, suggesting that either HK1 or HK2 is necessary for energetic metabolism. In addition, HK1 knockdown induced the EMT phenotype and accelerated tumor malignancy; in contrast, HK2 silencing did not cause any morphological change and did not affect cancer cell growth and migration. Furthermore, altered energy metabolism was observed in HK1-knocked down cells, but no particular energetic aberrations were detected in HK2-silenced cells (data not shown). These results indicate that HK2 overexpression induced by HK1 knockdown may contribute to the EMT phenotype and aberrant energy metabolism. To elucidate the functional role of HK2 overproduction in HK1 knocked down cells, it is necessary to either perform co-knockdown HK2 in HK1-silenced cells or to overproduce HK2 in mock cells. In HeLa cells, HK2 overexpression recapitulated the energetic alterations induced by HK1 silencing but only slightly increased cell growth, suggesting that simple HK2 overproduction cannot fully phenocopy the effects caused by HK1 knockdown. In addition, these results also indicate that both HK1 and HK2 cannot totally replace each other, and also suggest that both HK1 and HK2 indeed play different roles in energetic metabolism.

Cellular ATP is produced mainly by two metabolic pathways, glycolysis and aerobic respiration [54]. The energy yield (two ATP per glucose) from glycolytic metabolism is far lower than that from the respiratory pathway (about 30 ATP per glucose) [11]. As compared to the mock and vector-transfected cells, HK1-inhibited cells displayed a normal ATP level but this was accompanied by greatly elevated AMPK/p38 MAPK activity, indicating that HK1 knockdown resulted in either the dysfunction of aerobic respiration or a very low ATP/AMP ratio. In accordance with these results, HK1-silenced cells had a very low level of Δψ_m_ and ROS as compared with the mock and vector-transfected cells. These results suggest that either HK2 overexpression in HK1-knocked down cells cannot wholly fulfil the function of HK1 in the aerobic respiration or that both HK1 and HK2 play different roles and are involved in distinct energetic pathways such as aerobic respiration and glycolysis. For instance, the efficiency of G-6-P formation catalysed by HK1 and HK2 is different [12]. HK1-generated G-6-P may be predominantly used in aerobic respiration; in contrast, HK2-generated G-6-P may be mainly utilised in glycolysis. Because HK1 silencing decreased the G-6-P level, resulting in respiratory impairment, elevated HK2 expression either, induced by HK1 knockdown or caused by HK2 overproduction, increased G-6-P formation and in turn accelerated glycolytic metabolism, which could not fully compensate aerobic respiration.

Glucose is imported into cells by glucose transporters Gluts and is phosphorylated by HKs to generate G-6-P, which is subsequently used in either glycolysis for ATP formation or in the pentose phosphate pathway for metabolite synthesis [10, 11]. It has been shown that both glucose phosphorylation and mitochondrial binding are required for the protective effects of HK1 and HK2 against apoptosis [21]. Thus, the activity of HKs plays an important role in regulating cell survival and growth; the expression level of HKs must be under control to provide a normal supply of G-6-P. In addition, the analysis of both HK1 and HK2 expression revealed a high-low reciprocal pattern in many normal and cancerous cells, suggesting that their expression are homeostatically regulated to maintain total HK activity or G-6-P quantity. Furthermore, to preliminarily evaluate the role of HK1 and HK2 in tumor progression, expression of HK1 and HK2 was searched and analyzed within the Human Protein Atlas (http://www.proteinatlas.org/) website. The results revealed that most cancer types exhibit low to moderate levels of the HK1 protein but display moderate to high levels of HK2 protein (data not shown). Moreover, a recent study indicated that glioblastoma multiforme, the most common deadly malignant brain tumor, shows increased HK2 expression and downregulated HK1, whereas low-grade glioma and normal brain counterparts predominantly express HK1 [55]. Particularly, the malignant brain tumors rely mainly on aerobic glycolysis; in contrast, the normal brain tissues preferentially undergo oxidative phosphorylation. Consistent with these reports, this study revealed that HK1 knockdown not only increased HK2 expression but also upregulated glycolytic activity, suggesting that the function of HK1 is mainly involved in aerobic respiration, whereas HK2 plays a major role in glycolysis. However, the regulation of both HK1 and HK2 expression has still not been fully elucidated or investigated yet. To understand the role and function of both HK1 and HK2 in aerobic respiration and glycolysis, it is important to study the molecular mechanisms that are used to control or coordinate their expression and activities in these two metabolic pathways.

It has been shown that elevated glycolysis can be induced by a mitochondrial defect, hypoxic environment, oncogenic signal or enzymatic alteration in tumor cells [34]. Recently, we have reported that loss of the respiratory enzyme CS induces an EMT phenotype and alters energy metabolism from aerobic respiration to glycolysis, and results in accelerated cancer cell growth and metastasis [44]. In addition, malignant progression is due to the activation of EMT-related regulators; altered energy metabolism is caused by deregulation of the p53/TIGAR and SCO2 pathways. In this study, we demonstrated that HK1 knockdown not only impairs energetic metabolism, including respiratory dysfunction and increased glycolysis, but also causes an EMT switch and consequently accelerates tumor malignancy. These results reveal that both CS and HK1-silenced cells display almost identical phenotypes, including cellular and biochemical behaviors (except for ATP levels), suggesting that HK1-generated G-6-P may mainly fuel aerobic respiration and HK2-generated G-6-P may primarily couple to the glycolytic pathway. Furthermore, alterations in energetic metabolism induced by HK1 knockdown were almost recapitulated by HK2 overexpression, indicating that HK2 overproduction indeed contributes to these metabolic changes.

In recent years, there have been significant advances in cancer therapy, particularly targeting the intrinsic pathways or extrinsic mediators in tumor cells. The heavier dependence of cancer cells than of normal counterparts on glycolysis for ATP production offers a possibility for antitumor therapy: glycolytic inhibitors might target cancer cells by blocking energetic metabolism. Since HK1 knockdown induces tumor malignancy via deregulation of energetic metabolism from aerobic respiration to glycolysis, the commonly used glycolytic inhibitor 2-DG is thus a potential chemotherapeutic agent for HK1-silenced cells. In accordance with this speculation, HK1-knocked down cells indeed displayed not only a strongly glucose-dependent proliferation but also considerable 2-DG-induced growth inhibition as compared with the mock and vector-transfected cells. In addition, both glucose depletion and 2-DG administration induced apoptosis in HK1-knocked down cells. These results demonstrate that malignant tumors with increased glycolytic activity can be specifically targeted by glycolytic inhibitors.

Taken together, this study demonstrates that knockdown of the glycolytic enzyme HK1 but not HK2 results in the deregulation of energetic metabolism via increased HK2 expression and accelerates tumor malignancy through inducing an EMT phenotype. In addition, this work also suggests that glycolytic inhibitors should be used only to treat cancers with elevated glycolytic activity. Moreover, the HK1-silenced cells established in this study provide as a tool for screening or assessing potential anticancer drugs that specifically target the glycolytic pathway.

## MATERIALS AND METHODS

### Cell culture

Human cervical carcinoma derived HeLa and SiHa cells, human breast cancer derived MCF7 cells, and human prostate cancer derived PC3 cells were purchased from the American Type Culture Collection (ATCC; Rockville, MD, USA). They were grown and maintained in Dulbecco’s Modified Eagle’s Medium (DMEM; GIBCO BRL, Gaithersburg, MD, USA) supplemented with 10% heat inactivated foetal calf serum (FCS; Biological Industries, Kibbutz Beit Haemek, Israel) and 1% antibiotic/antimycotic solution (GIBCO BRL) at 37°C in a humidified incubator with 5% CO_2_. HK1 and HK2-silenced cells induced by shHK1-1 and shHK1-3 as well as shHK2-1 and shHK2-4 expression constructs were cultured and kept in growth medium containing 100 μg/ml hygromycin B. HK2-overexpressing cells generated by HK2 expression vector were grown and maintained in culture medium containing 100 μg/ml G418. These cells were subcultured several times a week after treatment with 0.1% trypsin (Biowhittaker Acambrex, Walkersville, MD, USA).

### Design and construction of shRNA expression vectors

Plasmid vectors or constructs were prepared according to standard molecular cloning techniques. The oligonucleotides used in this study were obtained from local commercial suppliers. The stable shRNA expression vector pSUPER/Hyg^r^ was constructed as described previously by subcloning the hygromycin resistance gene expression cassette (Hyg^r^) prepared from pDsRed2-N1 into the pSUPER [44, 56]. To design an effective shRNA expression vector, shRNAs were selected and constructed using a fully robust and comparative siRNA validation system [57]. In general, shRNA expression vectors were cloned by ligating an annealed oligonucleotide duplex into restriction enzyme *Bgl*II/*Hin*dIII sites in the pSUPER/Hyg^r^ vector. Designed and selected shHK1s and shHK2s that had efficiently knocked down HK1 and HK2 expression are shown in Supplementary Figure 1.

### Cell transfection

Cells were seeded in 6-well plates with a glass coverslip in each well at a density of 1 × 10^5^ cells per well. After 24 h, cells were transfected with 2 μg of shRNA expression constructs using Lipofectamine 2000 (Invitrogen, Carlsbad, CA, USA) according to the manufacturer’s instructions. At 48 h or various time periods after transfection, transfected cells were analysed by Western blotting, immunofluorescent staining or microscopic imaging. For clones with stable shHK1s or shHK2s expression, transfected cells were collected at 48 h after transfection, replated into 10 cm Petri dishes, and then selected in growth medium containing 300 μg/ml hygromycin B for about two weeks. Hygromycin B resistant colonies were isolated and recloned for further analysis and characterisation. The human HK2 (pWZL Neo Myr Flag HK2) expression vector was purchased from Addgene (Cambridge, MA, USA). For clones with stable HK2 expression, transfected cells were harvested at 48 hours after transfection, reseeded into 10 cm Petri dishes, and then screened in culture medium containing 400 μg/ml G418 for about two weeks. G418 resistant colonies were picked and recloned for further analysis and characterisation.

### Western blot analysis

Cells were collected by scraping at 4°C and dissolved in RIPA lysis buffer (150 mM NaCl, 50 mM Tris, pH 8.0, 0.1% SDS, 0.5% sodium deoxycholate, 1% NP-40) containing protease inhibitors (Roche Molecular Biochemicals, Mannheim, Germany). Total protein extracts or subcellular protein extracts prepared from mitochondrial and cytosolic fractions were electrophoresed on 12% SDS-PAGE and then electroblotted onto an Immobilon-P membrane (Millipore, Billerica, MA, USA). Blotted membranes were probed with mouse monoclonal antibodies specific for HK1 (G-1; Santa Cruz Biotechnology, Santa Cruz, CA, USA), β-actin (Sigma-Aldrich, Saint Louis, MO, USA), E-cadherin (BD Biosciences, San Jose, CA, USA), vimentin (V9, Sigma-Aldrich Chemical), α-SMA (1A4, Sigma-Aldrich Chemical) and Glut-3 (G-5; Santa Cruz Biotechnology); rabbit monoclonal antibody specific for AMPKα1 (Y365; Abcam, Cambridge, UK); rabbit polyclonal antibodies specific for IDH3A (Aviva Systems Biology, San Diego, CA, USA), p-AMPKα1 (phospho Thr 172; Abcam), Snail (H-130; Santa Cruz Biotechnology), Twist (H-81; Santa Cruz Biotechnology) and Glut-1 (H-43; Santa Cruz Biotechnology); goat polyclonal antibodies specific for OGDH (α-KGD, C-20; Santa Cruz Biotechnology), HK2 (C-14; Santa Cruz Biotechnology); sheep polyclonal antibody specific for LDH1 (431.1, Santa Cruz Biotechnology). Subsequently, the membranes were incubated with horseradish-peroxidase (HRP) conjugated goat anti-mouse IgG (H+L) (Pierce Biotechnology, Rockford, IL, USA), goat anti-rabbit IgG-HRP (Santa Cruz Biotechnology), donkey anti-goat IgG-HRP (Santa Cruz Biotechnology), or rabbit anti-sheep IgG-H&L-HRP (Abcam) secondary antibodies. Bands recognised by specific antibodies were visualised using an enhanced chemiluminescence system (Amersham Biosciences, Little Chalfont, Buckinghamshire, UK) according to the manufacturer’s protocols. Subcellular protein extracts of mitochondrial and cytosolic fractions were isolated as described by Yadava et al. (2004) [58].

### Immunofluorescent staining

Cells were grown on glass coverslips in 6-well plates for 48 h, then fixed with 3% paraformaldehyde and permeabilised with 0.2% Triton X-100. Immunofluorescent staining was performed according to standard protocols. Treated cells were first probed with anti-HK1, anti-HK2, anti-E-cadherin or anti-vimentin antibody as described above, and then incubated with Alexa Fluor 488 conjugated goat anti-mouse IgG (H+L) (Invitrogen) or FITC conjugated donkey anti-goat IgG (Santa Cruz Biotechnology) secondary antibody, as well as counterstained nuclei with DAPI. For stress fibre F-actin staining, prepared cells were directly stained with Alexa Fluor 488-conjugated phalloidin solution (Molecular Probes, Eugene, OR, USA). Stained cells were examined and imaged using an inverted fluorescence microscope (Olympus IX71; Olympus Co., Tokyo, Japan).

### Wound healing migration assay

Cells were grown on 6-well plates until confluent and then a line was scratched in the middle of well using a sterile plastic 1 ml micropipette tip. Cells were incubated in culture medium for various time periods and assessed with a time interval of 12 h. At each measured time point, wounds were imaged using an inverted phase-contrast microscope.

### Boyden chamber migration assay

Cells were cultured in 10 cm Petri dishes until 70-80% confluent and harvested by treatment with 0.1% trypsin (Biowhittaker Acambrex). Cells were plated onto 8 μm pore size polycarbonate filters (Nucleopore Corp., Pleasanton, CA, USA) in a 48-well Boyden chamber (Neuro Probe Inc., Gaithersburg, MD, USA) at 2.5 × 10^4^ cells per well and incubated for 6 h. Chemotactic migration of cells was induced by 10% FCS in the lower chamber. The cells that migrated were fixed with 100% ethanol and then stained with Liu’s staining solution. Stained cells were imaged and examined under an inverted phase-contrast microscope.

### Matrigel invasion assay

Cells were grown in 10 cm Petri dishes until 70-80% confluent and collected by trypsinisation with 0.1% trypsin (Biowhittaker Acambrex). Cells were seeded into an 8 μm invasion chamber at a density of 1 × 10^5^ cells per well and incubated for 8 h. Chemotactic invasion of cells was induced by 10% FCS in the lower chamber. The invasive cells were fixed with 100% ethanol and then stained with GIMSA staining solution. Stained cells were imaged and analysed under an inverted phase-contrast microscope.

### Methyl thiazolyl tetrazolium (MTT) assay

Cells were seeded into 96-well plates at 1 × 10^4^ cells per well in 200 μl culture medium. At 6 h before each examined time point, except day zero (Day 0), cells were treated with 10 μl of MTT solution (5 mg/ml) for 6 h, which was then dissolved in 200 μl of dimethyl sulfoxide (DMSO) and detected at 590 nm using an ELISA reader (VERSA_max_ tunable microplate reader, Molecular Dynamics, Sunnyvale, CA, USA).

### Bromodeoxyuridine (BrdU) incorporation assay

Cells were cultured in 96-well plates at a density of 1 × 10^4^ cells per well in 200 μl growth medium. After 46 h of incubation, cells were treated with BrdU for 2 h, then fixed and the BrdU (colorimetric) cell proliferation ELISA was carried out (Roche Diagnostics GmbH, Penzberg, Germany) following the manufacturer’s instructions.

### Colony formation assay

Cells were seeded in 6-well plates at 2 × 10^3^ cells per well. After 6 or 10 days, colonies were stained with crystal violet for 24 h, then rinsed with deionised distilled water, imaged and scored using a Nikon D80 digital camera (10 Mega-pixel; Nikon Corp., Tokyo, Japan) and AlphaEase FC software (Alpha Innotech Inc., San Leandro, CA, USA), respectively.

### Soft agar colony formation assay

Soft agar colony formation assay was carried out in 6-well plates at a density of 5 × 10^3^ cells per well. Cells mixed in 1.5 ml of top agar (0.35% agar in growth medium) were overlaid onto a layer of 1.5 ml of bottom agar (0.7% agar in the same culture medium). After 12 days, colonies were imaged and scored using an inverted phase-contrast microscopy without fixation or staining. Colonies with more than 300 cells were scored.

### *In vivo* tumor growth analysis

Cells (1 × 10^5^) were subcutaneously inoculated into the back of NOD/SCID mice. Tumors were detected externally using Vernier callipers every 3 to 5 days. Tumor volume was calculated as length × width^2^ × 0.52. After 20 or 60 days, mice were anesthetised with pentobarbital and imaged, after which they were culled and the tumors removed and imaged. Eight- to 12-week-old female mice were used in all animal experiments. The methods used in animal studies were carried out in accordance with the relevant guidelines. All animal experiments were approved by the Institutional Animal Care and Use Committee (IACUC) of the National Cheng Kung University, Tainan, Taiwan. The IACUC approval number is 102108.

### *In vivo* tumor metastasis analysis

Cells (1 × 10^5^) were intravenously injected into the tail vein of NOD/SCID mice. After 20 days, mice injected with HK1 knockdown cells appeared to be sick, so all mice were culled and examined for tumor metastases. Their organs, particularly lungs and hearts, were removed and fixed in formalin, and processed for histological and immunohistochemical analyses.

### Histological and immunohistochemical analyses

Mouse organs, i.e. lungs and hearts, were embedded in paraffin blocks and processed into random sections 4 μm thick. Tissue sections were further stained with hematoxylin and eosin (H&E) or immunohistochemically probed with an antibody specific for vimentin (V9; DAKO, Glostrup, Denmark) and counterstained with hematoxylin. The antigen was visualised as a brown precipitate.

### Mitochondrial membrane potential (Δψ) assay

Cells were cultured in 6-well plates at 1 × 10^5^ cells per well. After 48 h, cells were loaded with 150 nM tetramethylrhodamine methyl ester (TMRM) for 30 min in the growth medium. Stained cells were imaged using an inverted fluorescence microscope and quantitatively analysed by flow cytometry using a FACSCalibur™ flow cytometer (Beckton Dickinson, San Jose, CA, USA).

### Reactive oxygen species (ROS) detection

Cells were grown in 6-well plates at a density of 1 × 10^5^ cells per well. After 48 h, cells were treated with 10 μM 5–6 chloromethyl-2′,7′-dichlorodihydro-fluorescein diacetate acetyl ester (CM-H_2_DCFDA; Molecular Probes Inc., Eugene, OR, USA) for 30 min in phenol red-free culture medium. Stained cells were imaged using an inverted fluorescence microscope and quantitatively measured by flow cytometry using a FACSCalibur™ flow cytometer (Beckton Dickinson).

### Firefly luciferase ATP assay

Total cellular ATP was measured using the ATP Bioluminescence Assay Kit CLSII (Roche Applied Science, Mannheim, Germany) according to the manufacturer’s instructions. Luminescent intensities were quantified using a bioluminescence detection system (Lumat LB 9507 luminometer, Berthold Technologies, Bad Wildbach, Germany).

### Glucose uptake assay

Cells were cultured in 6-well plates at 1 × 10^5^ cells per well. After 48 h, cells were pre-incubated in glucose-free Krebs-Ringer bicarbonate (KRB) buffer (129 mM NaCl, 5 mM NaHCO_3_, 4.8 mM KCl, 1.2 mM KH_2_PO_4_, 1.0 mM CaCl_2_, 1.2 mM MgSO_4_, 10 mM HEPES, and 0.1% BSA; pH 7.4) for 30 min and then incubated in fresh KRB buffer supplemented with 600 μM 2-[N-(7-nitrobenz-2- oxa-1,3-diazol-4-yl)amino]-2-deoxy-D-glucose (2-NDBG; Molecular Probes Inc.), a D-glucose fluorescent analogue, and 3.3 mM glucose for 10 and 30 min. Treated cells were imaged using an inverted fluorescence microscope and quantitatively measured by flow cytometry using a FACSCalibur™ flow cytometer (Beckton Dickinson).

### Lactate dehydrogenase activity assay

Cells were seeded into 96-well plates at a density of 1 × 10^4^ cells per well in 200 μl growth medium for 2 h. Cells were directly lysed, and lactate dehydrogenase activity was measured using the Lactate Dehydrogenase Activity Assay Kit (BioVision, Mountain View, CA, USA) according to the manufacturer’s instructions.

### pH measurement in conditioned medium

Cells were inoculated into 6-well plates at 1 × 10^5^ cells per well. Cells were grown until confluent and then incubated in fresh growth medium for 12 h. The color and pH value of the conditioned media were imaged using a Nikon D80 digital camera (Nikon Corp.) and measured using a FE20-FiveEasy™ pH meter (Mettler Toledo, Schwerzenbach, Switzerland), respectively.

### Propidium iodide and annexin V-FITC double staining assay

Cell death analysis was examined using the FITC Annexin V Apoptosis Detection Kit I (Beckton Dickinson) according to the manufacturer’s protocols. Stained cells were quantitatively analysed by flow cytometry using a FACSCalibur™ flow cytometer (Beckton Dickinson).

## SUPPLEMENTARY MATERIALS FIGURES


