# Supplementary material for: The decrease of glycolytic enzyme hexokinase 1 accelerates tumor malignancy via deregulating energy metabolism but sensitizes cancer cells to 2-deoxyglucose inhibition
Source: Oncotarget. 2018 Apr 10;9(27):18949–69. doi: 10.18632/oncotarget.24855 (PMC5922369; doi:10.18632/oncotarget.24855)
Supplement: Supplementary file 1 [file oncotarget-09-18949-s001.pdf]

The decrease of glycolytic enzyme hexokinase 1 accelerates tumor malignancy via deregulating energy metabolism but sensitizes cancer cells to 2-deoxyglucose inhibition

SUPPLEMENTARY MATERIALS

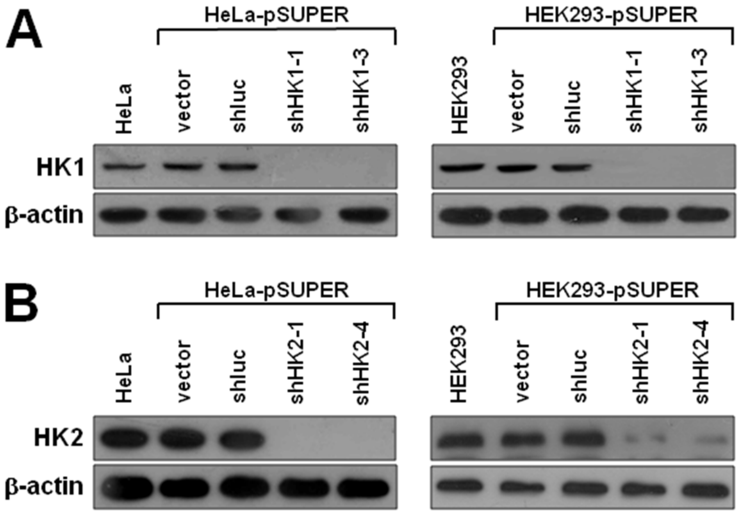

Supplementary Figure 1: Human HK1 and HK2 are knocked down efficiently by the RNAi technique. (A) Western blotting of HK1 in cells transfected with pshHK1 constructs. Total proteins isolated from transfected cells as indicated were blotted with antibodies for HK1 and β-actin. **(B)** Western blotting of HK2 in cells transfected with pshHK2 constructs. Total proteins isolated from transfected cells as indicated were blotted with antibodies for HK2 and β-actin. The β-actin level serves as the control for protein loading.

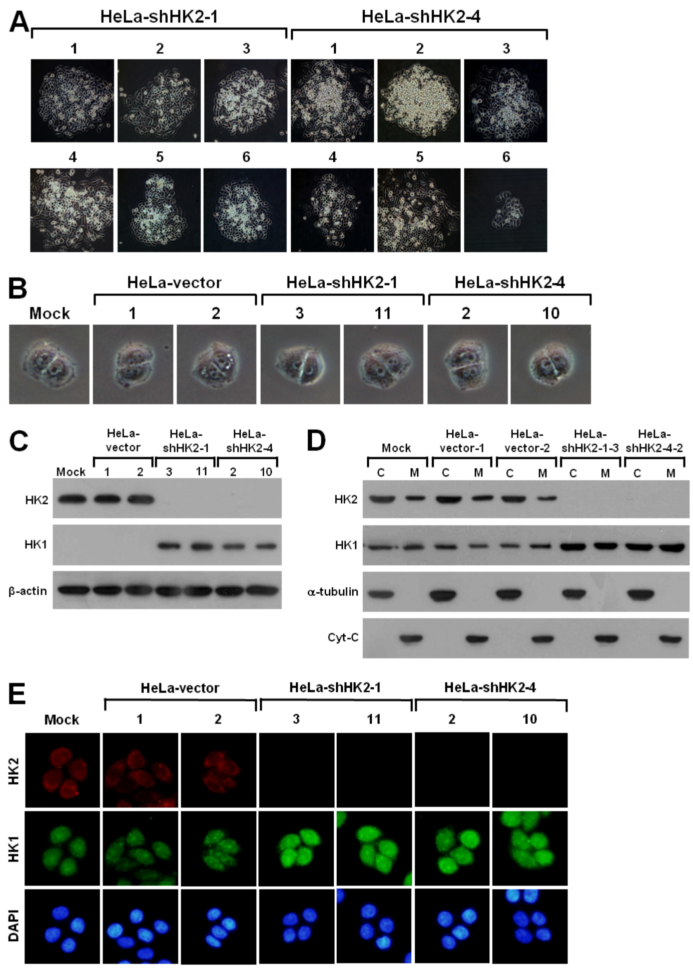

Supplementary Figure 2: HK2 knockdown does not induce a morphological change in human cervical cancer cells. **(A)** Colony morphology of shHK2-transfected cells. HeLa cells were first introduced with HK2 knockdown constructs (pshHK2-1 and pshHK2-4), and then selected for hygromycin resistant colonies. Colonies were picked up according to cell morphology and imaged. **(B)** Cell morphology of HK2-silenced cells. Four selected and validated HK2-knocked down, mock and two vector-transfected cells were compared regarding cell morphology. **(C)** Western blotting of HK1 and HK2 in HK2-knocked down cells. Total protein extracts prepared from cells as indicated were probed with antibodies specific for HK1, HK2 and β-actin. The β-actin level serves as the control for protein loading. **(D)** Western blotting of HK1 and HK2 in cellular fractions isolated from HK2-silenced cells. Cytosolic and mitochondrial extracts isolated from cells as indicated were probed with antibodies specific for proteins as labelled. The α-tubulin and cytochrome C (Cyt-C) serve as markers specific for the cytosol and mitochondria, respectively. **(E)** Immunofluorescent staining of HK1 and HK2 in HK2-knocked down cells. Cells as indicated were stained with antibodies specific for HK1 and HK2, as well as counterstained nuclei with DAPI.

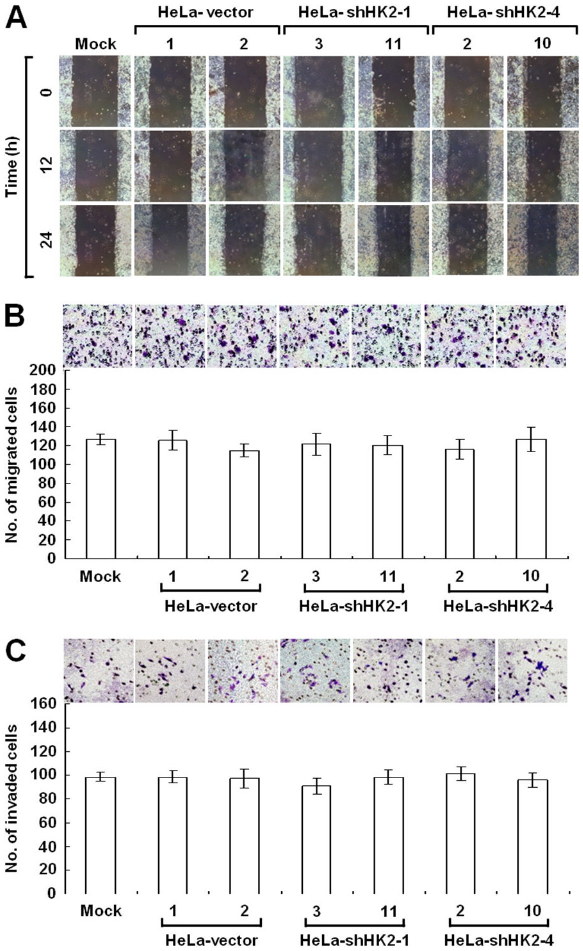

Supplementary Figure 3: HK2 inhibition does not affect tumour cell motility. (A) Wound healing migration assay of HK2-knocked down cells. Cells as indicated were cultured until confluent, then the wound healing migration assay was carried out and the wounds were imaged after incubation for various time periods as labelled. **(B)** Boyden chamber migration assay of HK2-silenced cells. Cells as indicated were loaded into Boyden chambers, then migrated chemotactically for 6 h. The cells that migrated were stained, imaged and enumerated. **(C)** Matrigel invasion assay of HK2-knocked down cells. Cells as indicated were seeded in invasion chambers, then invaded and moved chemotactically for 8 h. The cells that invaded were stained, imaged and enumerated. The plotted data are averaged from three independent experiments and the bars represent mean ± SD.

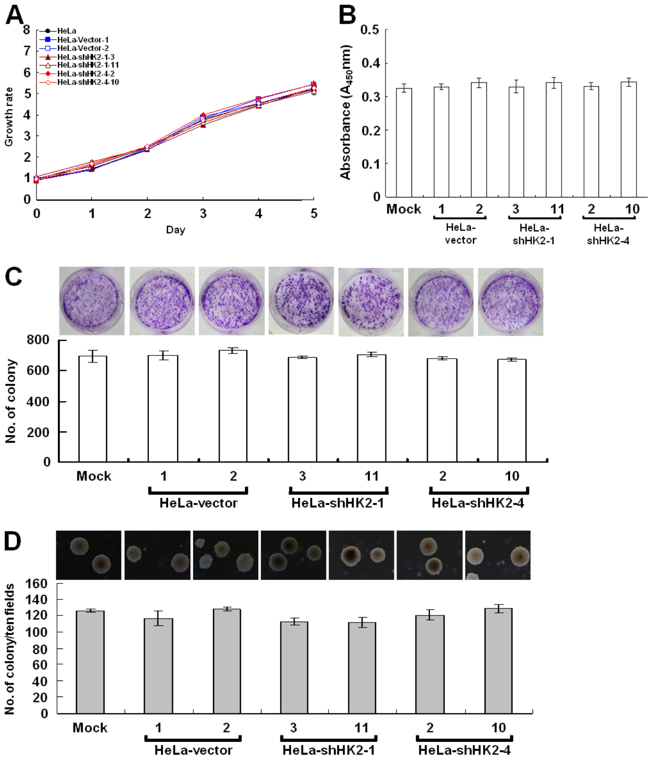

Supplementary Figure 4: HK2 silencing does not affect tumour cell growth. **(A)** MTT cell growth assay of HK2 knockdown cells. Cells as indicated were seeded in 96-well plates, incubated for various time periods as labelled, then the MTT cell growth assay carried out according to standard protocols. **(B)** BrdU incorporation assay of HK2 knockdown cells. Cells as indicated were loaded into 96-well plates and incubated for 3 days, then the BrdU (colorimetric) cell proliferation ELISA was carried out according to the manufacturer's procedures. **(C)** Colony formation assay of HK2 knockdown cells. Cells as indicated were cultured in 6-well plates for 6 days. Colonies were fixed, stained, imaged and enumerated. **(D)** Soft agar colony formation assay of HK2 knockdown cells. Cells as indicated were suspended in 0.35% top agar and overlaid on 0.7% bottom agar, and then cultured in 6-well plates for 12 days. Colonies were directly imaged and enumerated. The plotted data are averaged from three independent experiments and the bars represent mean ± SD.
